# Antioxidant Tocols as Radiation Countermeasures (Challenges to be Addressed to Use Tocols as Radiation Countermeasures in Humans)

**DOI:** 10.3390/antiox7020033

**Published:** 2018-02-23

**Authors:** Ujwani Nukala, Shraddha Thakkar, Kimberly J. Krager, Philip J. Breen, Cesar M. Compadre, Nukhet Aykin-Burns

**Affiliations:** 1 Department of Pharmaceutical Sciences, College of Pharmacy, University of Arkansas for Medical Sciences, Little Rock, AR 72205, USA; uxnukala@ualr.edu (U.N.); KJKrager@uams.edu (K.J.K.); BreenPhilipJ@uams.edu (P.J.B.); CompadreCesarM@uams.edu (C.M.C.); 2 Joint Bioinformatics Graduate Program, University of Arkansas at Little Rock, Little Rock, AR 72204, USA; 3 Division of Bioinformatics and Biostatistics, National Center for Toxicological Research, US Food and Drug Administration, Jefferson, AR 72079, USA; Shraddha.Thakkar@fda.hhs.gov; 4 Tocol Pharmaceuticals, LLC, Little Rock, AR 77205, USA

**Keywords:** tocopherol, tocotrienol, tocols, radioprotectors, radiation countermeasures, radiomitigators, alpha tocopherol transfer protein

## Abstract

Radiation countermeasures fall under three categories, radiation protectors, radiation mitigators, and radiation therapeutics. Radiation protectors are agents that are administered before radiation exposure to protect from radiation-induced injuries by numerous mechanisms, including scavenging free radicals that are generated by initial radiochemical events. Radiation mitigators are agents that are administered after the exposure of radiation but before the onset of symptoms by accelerating the recovery and repair from radiation-induced injuries. Whereas radiation therapeutic agents administered after the onset of symptoms act by regenerating the tissues that are injured by radiation. Vitamin E is an antioxidant that neutralizes free radicals generated by radiation exposure by donating H atoms. The vitamin E family consists of eight different vitamers, including four tocopherols and four tocotrienols. Though alpha-tocopherol was extensively studied in the past, tocotrienols have recently gained attention as radiation countermeasures. Despite several studies performed on tocotrienols, there is no clear evidence on the factors that are responsible for their superior radiation protection properties over tocopherols. Their absorption and bioavailability are also not well understood. In this review, we discuss tocopherol’s and tocotrienol’s efficacy as radiation countermeasures and identify the challenges to be addressed to develop them into radiation countermeasures for human use in the event of radiological emergencies.

## 1. Introduction

The use of ionizing radiation is increasing day by day for various purposes, including its clinical uses for diagnostic purposes and cancer treatment and in non-clinical applications, such as the nuclear generated energy production, engineering, construction, and sterilization of food products [1,2,3]. With such widespread uses, the likelihood of an intentional or unintentional encounter with radiation is quite high. The risk of radiation exposure has been increasing mainly with increased use of ionizing radiation for nuclear power plants or nuclear weapons, both of which can result in accidental radiological emergencies. There were nearly 105 civilian and military nuclear reactor accidents between 1952 and 2015 that resulted in massive loss of human life and property [1]. Recently, on 24 August 2017 the Pennsylvania Department of Health distributed potassium iodide tablets for free to the residents who live or work within 10 miles of the Peach Bottom and Three Mile Island nuclear plants to be ready in case of an emergency. However, potassium iodide, a specific blocker of thyroid radioactive iodine uptake, only protects the thyroid gland of individuals exposed to radiation.

Exposure to ionizing radiation produces oxygen derived reactive oxygen and nitrogen species (ROS and RNS), including hydroxyl radical (OH•), superoxide (O_2_•^−^), peroxynitrite (ONOO^−^), and hydrogen peroxide (H_2_O_2_). Ionizing radiation-induced ROS and RNS damage DNA, proteins, and lipids as well as activate intracellular signaling pathways and stimulate cytochrome C release from mitochondria, leading to apoptosis [4,5,6]. 

The Office of Science and Technology Policy and the United States Department of Homeland Security have identified radiation countermeasure development as the highest priority for preparedness against a potential bioterrorism event [1]. As of today, amifostine is the only FDA approved drug for use in patients undergoing radiotherapy. However, because of its adverse side effects, its use is limited and the search for safe and effective radiation countermeasures continues. 

Vitamin E and its derivatives have attracted the attention of researchers in recent years for their radioprotective effects, which have been heavily studied against total body irradiation [7,8,9,10], as well as partial body irradiation [11,12,13,14,15,16]. In this review, we outline the research endeavors dedicated to studying the radiation protection efficacy of vitamin E analogs. We also identify the issues that need to be addressed when using vitamin E analogues as safe and effective radiation countermeasures in humans.

## 2. Radiation Induced Injuries

Ionizing radiation is radiation that carries sufficient energy to liberate electrons from atoms or molecules leaving them with unpaired electrons thereby ionizing them and producing free radicals. These free radicals and ROS/RNS including, OH•, O_2_•^−^, ONOO^−^, and H_2_O_2_ can damage nucleic acids, proteins, and membrane lipids. Exposing an individual to ionizing radiation for a brief period can cause severe tissue injuries, referred to as Acute Radiation Syndrome (ARS). ARS can occur with doses higher than 1 Gray (Gy) that are delivered at relatively high rates. The three clinical syndromes of ARS are based on the acute whole-body dose, duration, and dose rate, including hematopoietic or bone marrow sub-syndrome, gastro-intestinal sub-syndrome, and cerebrovascular sub-syndrome. Each of these three sub-syndromes follows a 4-phase clinical pattern according to CDC Emergency Preparedness and Response, as detailed in Table 1 [17]

## 3. Radiation Countermeasures

Effective radiation countermeasures should be safe, efficient, stable, easy to administer, and have good bioavailability. Radiation countermeasures fall under three categories, radiation protectors, radiation mitigators, and radiation therapeutics. 

Radiation protectors are agents that are administered prior to radiation exposure to protect from radiation-induced injuries by many mechanisms, such as scavenging free radicals that are generated by initial radiochemical events, delaying cell cycle, promoting DNA repair, etc. Radiation protectants are given to personnel that are at risk of exposure to radiation like the military, first responders and civilians during the evacuation of disaster areas. Radiation mitigators are agents that are administered after the exposure of radiation but before the onset of symptoms. They accelerate the recovery and repair from radiation-induced injuries. Whereas radiation therapeutic agents are administered after the onset of symptoms and act by regenerating the tissues that are injured by radiation. Radiation mitigators and radiation therapeutics can be given to people who are victims of nuclear accidents or terrorist attacks or to patients undergoing radiotherapy. One among the various candidates [18,19,20,21] that are under development as radiation countermeasures are vitamin E’s tocols.

Natural products with health benefits are often attractive targets for research [22]. Among the natural products, vitamins are notably considered beneficial for human health. Vitamin E is a well-known antioxidant that can scavenge free radicals produced by radiation exposure. Vitamin E is found in our diet and has an acceptable toxicity profile [23]. 

**Figure 1:**
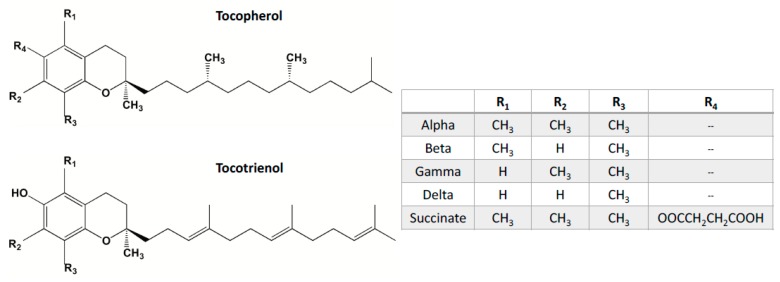
Chemical structures of tocopherol and tocotrienol isoforms.

The vitamin E family consists of eight different naturally occurring vitamers, four saturated analogs (α, β, γ, and δ) called tocopherols, and four unsaturated analogs (α, β, γ, and δ) referred to as tocotrienols, which are collectively called tocols. Tocopherols and tocotrienols are structurally similar with the same chromanol head, except that tocotrienols have unsaturated farnesyl isoprenoid side chain at C-3′, C-7′, and C-11′, whereas tocopherols have saturated phytyl isoprenoid side chain (Figure 1).

### 3.1. Tocopherols and Tocopherol Succinate

Vitamin E components have been reported to be radioprotective in various studies [24,25,26]. However, there are several factors that influence the differential radioprotective efficacy of tocol analogs, including the rate of absorption after oral administration, which is found to be greater in tocotrienols than tocopherols due to higher absorption of tocotrienols by the intestinal epithelial cells [27]. Serbinova et al. [28] reported that the antioxidant potential of tocotrienols is 1600 times more than that of α-tocopherol (AT). Studies by Pearce et al. [29] and Qureshi et al. [30] suggest that tocotrienols are better radio-protectants because of their ability to inhibit HMG-CoA reductase.

AT’s dose reduction factor was determined to be 1.11 when administered subcutaneously at a dose of 100 IU/kg when administered within 15 m after irradiation of 9 Gy in mice. AT significantly increased the 30-day survival of male CD2F1 mice when given one h before or within 15 m after irradiation. Combination studies of α-tocopherol with WR-3689 showed that the radio-protective efficacy of WR-3689 (150 mg/kg) significantly increased when given in combination with α-tocopherol at 100 IU/kg with a dose reduction factor of 1.49 [31]. AT also reduced the frequency of micronuclei and chromosomal aberrations in bone marrow cells when administered orally either 2 h before, immediately after, or 2 h after irradiation of 1 Gy in mice [32]. The study by Kumar et al. [33], demonstrated that AT has more radio-protective activity when administered subcutaneously than when given orally at a dose of 400 IU/kg 24 h before total body irradiation at 10.5 Gy. AT (20 IU/kg/day) in combination with pentoxifylline (100 mg/kg/day) induced a significant improvement in radiation-induced myocardial fibrosis and left ventricular diastolic dysfunction after irradiation at 9 Gy [34]. A study by Empey et al. [35] showed that AT protects gastrointestinal mucosa against radiation induced absorptive injury when administered before 10 Gy of abdominal radiation. In another study, AT protected mice from radiation injury when administered i.p. after irradiation of <10 Gy, when administered no later than 5 h after irradiation, suggesting enhancement of repair processes and antioxidant scavenging of metabolically produced radicals when produced by irradiation [36]. 

To study the role of hematopoietic cytokines in the radioprotective activity of tocopherol succinate and other tocols (α-tocopherol, δ-tocopherol, γ-tocopherol, γ-tocotrienol, and tocopherol acetate), Singh et al. [8] measured cytokine levels by Luminex, ELISA, and cytokine array in mice serum after administrating 400 mg/kg tocols subcutaneously 24 h before whole-body irradiation at a dose of 3 and 7 Gy. Among all the tocols studied, tocopherol succinate was most effective in stimulating granulocyte colony stimulating factor (G-CSF) and IL-6. Since G-CSF and IL-6 play an important role in hematopoietic injury, the study indicates that tocopherol succinate’s radio protective activity is mediated by cytokines [8]. When tocopherol succinate injected mice were administered a neutralizing antibody to G-CSF, the protective effect of tocopherol succinate was significantly abrogated [9]. A study by Singh et al. [10] showed that tocopherol succinate protects mice against lethal doses of ionizing radiation by inhibiting radiation-induced apoptosis and DNA damage as well as by increasing cell proliferation. In a study by Singh et al. [37], the dose reduction factor of tocopherol succinate was determined to be 1.28 in mice, administered subcutaneously with 400 mg/kg of tocopherol succinate, 24 h before total body irradiation at 9, 9.5, 10, 10.5, 10.75, and 11 Gy. This study showed that tocopherol succinate stimulated high levels of G-CSF with a peak at 24 h, moderate levels of IL-6 between 24 and 48 h after treatment, and protected myeloid components from radiation injury at 3 and 7 Gy. A similar study [38] suggests that tocopherol succinate protects mice from radiation-induced gastrointestinal damage by promoting the generation of crypt cells and inhibiting apoptosis and translocation of gut bacteria to the heart, spleen, and liver in irradiated mice. Tocopherol succinate has also demonstrated radioprotection from total body irradiation by decreasing the number of CD68-positive cells, reducing DNA damage, and apoptotic cells and by increasing proliferating cells in irradiated mice [10]. Tocopherol succinate also modulates the expression of antioxidant enzymes and inhibits expression of oncogenes in irradiated mice, according to a study by Singh et al. [39].

### 3.2. Tocotrienols

Multiple studies have reported the antioxidant, anti-inflammatory, anticancer, hypocholesterolemic, and neuroprotective properties of tocotrienols in different cell lines, animal models, and in humans. This review will discuss their radioprotective activity studied in different animal models and in humans.

A number of studies have shown that tocotrienols are superior antioxidants compared to tocopherols [28,29,40,41,42,43]. Studies [24,44] have demonstrated that γ-tocotrienol (GT3) protects against radiation injury by increasing hematopoietic progenitors, neutrophils, platelets, white blood cells, and reticulocytes. Singh et al. [45] evaluated the protective effects of GT3 in nonhuman primates treated with 5.8, 6.5, and 7.2 Gy doses of cobalt-60 gamma radiation (0.6 Gy/min). This study reports the pharmacokinetic (PK) parameters (at 9.375, 18.75 and 37.5 mg/kg doses) and efficacy of GT3 (37.5 mg/kg and 75 mg/kg). Their PK analysis showed increased area under the curve with increasing drug dose and half-life of GT3. Unexpectedly, *t*_max_ increased in a dose-dependent manner. This could be due to the slow release of GT3 from the site of injection (sub-cutaneous). The study also demonstrated that GT3’s efficacy in reducing the severity of neutropenia and thrombocytopenia is dose-dependent, and 75 mg/kg treatment is more effective than 37.5 mg/kg treatment after a 5.8 Gy dose of ionizing radiation. However, there was no significant difference in animal survival at 60 days between the vehicle group and the GT3 treated groups. 

The study by Li et al. [46] demonstrated that a single injection of δ-tocotrienol (DT3), given to mice 24 h before a total body irradiation, presented 100% survival, measured by 30-day post-irradiated survival, by increasing cell survival and regeneration of hematopoietic microfoci. Singh et al. [43] showed evidence that DT3 mediates its radioprotection by inducing G-CSF in irradiated mice and also showed that DT3 induces high levels of several cytokines, comparable to other tocols in mice. A study by Loose et al. [47] suggested that GT3 is more efficacious than the tocopherols in terms of radiation protection because of its greater potency to induce gene expression in human endothelial cells. This could be due to the low cellular uptake of α-tocopherol compared to GT3. When GT3 was administered s.c. at 200 mg/kg in combination with pentoxifylline, showed synergistic radioprotection activity in mice exposed to 11.5 Gy total body irradiation, and this radioprotective activity of GT3 was shown to be mediated through induction of G-CSF [48]. 

Studies by Satyamitra et al. [23] showed that DT3 has radiation protection and mitigation effects. DT3, when given s.c. at 150 mg/kg or 300 mg/kg 24 h before total body irradiation, showed effective radioprotection in mice. In addition, DT3 showed reduced lethality when administered at 150 mg/kg in mice 2, 6, or 12 h after irradiation. Another study by Kumar et al. [49] indicated that a GT3 and DT3 combination at 800 mg/kg given orally twice a day for six months to patients who had radiotherapy for head and neck cancer had significantly improved mouth opening and subjective symptoms due to radiation-induced fibrosis. Studies also suggested that tocols exert their biological effects not only by their antioxidant properties but also by inhibiting HMG-CoA reductase [49]. Tocotrienols accumulate in the small intestine as well as in the colon to a greater level than tocopherols, and this may aid with their ability to reduce GI injury [50]. A study by Naito et al. [51] demonstrated that GT3 concentrations in endothelial cells were 30–50 times greater than those of α-tocopherol. The effect of GT3 on tetrahydrobiopterin (BH4) bioavailability was studied by Berbee et al. [52], where GT3 counteracted the decrease in BH4. GT3 protected hematopoietic tissue by preserving the hematopoietic stem cells (HSCs) and hematopoietic progenitor cells (HPCs). The HPC numbers in GT3 treated mice recovered 90% at day seven after total body irradiation [44]. Modulation of sphingolipids leads to cellular stress and upregulation of A20, a well-established NF-kB negative regulator. It has been shown that the mechanism of GT3 radioprotection may involve the inhibition of NF-κB activation by induction of A20 [53,54]. It also has been shown that the ability of GT3 to protect against vascular injury is related to its ability to inhibit HMG-CoA reductase [55].

## 4. Vitamin E Tocols 

Vitamin E in the diet passes through the gastrointestinal tract and gets absorbed in the small intestine. It is emulsified by bile and absorbed in the form of micelles [56]. Yap et al. [57] have reported that the dietary fat consumption plays an important role in the absorption of tocols. The administration route of vitamin E also influences its absorption [58,59]. 

### 4.1. α-Tocopherol Transfer Protein

**Figure 2:**
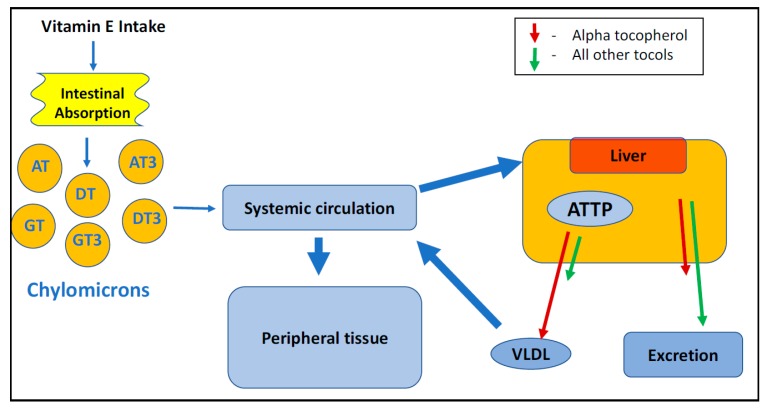
Major pathway for vitamin E absorption and metabolism.

The circulating levels of vitamin E in the body are maintained by α-tocopherol transfer protein (ATTP), which is abundantly found in the liver. This hepatic protein is a member of a lipid-binding protein family and plays a major discriminating role in the plasma and tissue retention of dietary tocols. This protein is regulated by α-tocopherol transfer gene present on chromosome 8q13 [60]. ATTP has two different conformations, closed and open [61]. The structure of ATTP has a hinge, which flips to a closed conformation after incorporating AT in the binding pocket. The fate of the tocol depends on the preferential binding of ATTP in the liver, and those that are not selected by ATTP are excreted via bile or renal excretion (Figure 2) [25,61]. ATTP has a much greater affinity AT than for all other tocols, and this would explain why the other tocols exhibit lower plasma levels over time [62,63,64]. Vitamin E supplements are commonly formulated with AT, which is often present as a synthetic racemic mixture. However, only the naturally occurring RRR isomer of AT has a high affinity for ATTP, causing the other isomers to be much less bioavailable [65]. Studies [25,66] have proposed that unsaturation in the side chain of tocotrienols accounts for their lower affinity for ATTP by making it impossible for the tocotrienol bend inside the ligand-binding pocket of ATTP and hindering the protein’s ability to attain the closed conformation. Thus, there is a significant difference in the distribution and metabolism of tocols among various tissues [67,68,69,70]. 

There is a population, with a neurodegenerative disease known as ataxia, with vitamin E deficiency (AVED) [71]. Patients diagnosed with AVED have three frame shift mutations in the α-tocopherol transfer gene present on chromosome 8q13. AVED is an autosomal recessive defect in which the patient is not able to absorb vitamin E into the systemic circulation and becomes deficient [71]. Without a regular supply of this key antioxidant, the body becomes susceptible to damage by free radicals. The coordination of movement becomes uncontrolled and the patient experiences a loss of sensation [71]. Individuals suffering from AVED have normal intestinal absorption of vitamin E, but they are unable to maintain normal levels of vitamin E in systemic circulation. The reason for this is that AVED patients lack functional ATTP, thus the recirculation of α-tocopherol is inhibited. To maintain a normal blood concentration of vitamin E, patients suffering from AVED require very high doses of oral vitamin E throughout their lives [71]. AVED exemplifies the importance of the presence of functional ATTP. However, the fact that ATTP has a much greater affinity for α-tocopherol than for other tocols results in a much faster elimination rate and lower plasma levels over time for all the other tocopherols and for tocotrienols.

### 4.2. Absorption and Distribution

It is likely that the inconclusive outcomes of various vitamin E clinical trials may be due to limited understanding of the pharmacokinetics of tocols. The bioavailability of vitamin E in humans is dependent on many factors. Studies have shown that vitamin E bioavailability is greater when administered with food, particularly fat [57,58]. Reboul et al. [72] studied the role of the SR-B1 receptor in the intestinal absorption of vitamin E and showed that the expression of the SR-B1 receptor may be responsible for the inter-individual variability in vitamin E absorption and that this receptor mediates the intestinal vitamin E absorption. 

Though there are several studies showing tocotrienol’s radioprotective activity, their dosing, efficacy, and therapeutic concentrations still remain controversial. This is partially due to the lack of sufficient data explaining their absorption, distribution, and elimination. There is a large variability in the rate and extent of absorption of tocotrienols in different populations from healthy subjects to smokers and diseased patients. The route of administration can also play an important role in the plasma concentrations of tocotrienols [45,57,58]. 

The absorption of tocotrienols was negligible when administered via intraperitoneal and intramuscular route, whereas the absorption was incomplete when given orally [58]. Several studies have focused on developing a water-soluble delivery system to increase the solubility of the lipophilic tocols. The use of cyclodextrin and self-emulsifying formulations showed improved absorption and higher plasma levels in rats. The peak plasma concentration (*C*_max_) and the area under the curve (AUC) of tocotrienols administered with self-emulsifying systems were increased 2–4-fold compared to non-emulsified formulations [73,74]. In a study by Yap et al. [57], comparisons were made between eight healthy male volunteers given 300 mg mixed tocotrienols (87 mg α-tocotrienol, 166 mg GT3, and 43 mg DT3) under a fasted state or after a fatty meal. The 24 h AUC of tocotrienols was increased by at least 2-fold in the fed state. The maximum plasma concentrations for α, γ, and δ tocotrienols were found to be 1.83, 2.13, and 0.34 µg/mL, respectively. While in another study, where tocotrienols were given at higher doses of 296 mg α-tocotrienol, 284 mg GT3, and 83 mg DT3, the maximum plasma concentrations were 1.55, 2.79, and 0.44 µg/mL, respectively [75]. The discrepancies in the plasma levels observed may be an indication that levels may depend on the ratios of the analogs [76]. It has been reported that tocotrienols were transported in triacylglycerol-rich fractions after administration of tocotrienol-rich fraction (TRF) at 1011 mg in healthy subjects, and tocotrienols were found in significant amounts in the plasma and lipoproteins [75]. This study [75], as well as other studies [77,78,79], indicate that high TRF doses from 200 to 3200 mg/d are safe for healthy human consumption. However, it was also mentioned in a review by Ju et al. [80] that the therapeutic efficacy of tocotrienols depends on dose, formulation, route of administration, and study population. Several studies [81,82,83,84] investigated the changes in lipid profile at different doses of tocotrienols. 

In spite of all these studies, the dose dependent effect of TRF on the lipid profile is still inconclusive, and further studies are needed to identify the factors responsible for this disparity. For example, it was reported that reduced DNA damage was observed in people over the age of 50 years who were given tocotrienols [85]. This was supported by a later study by Chen et al. [86] who compared the absorption of tocotrienols in different age groups of subjects who were given TRF supplementations for six months. They observed that the plasma tocotrienol levels increased significantly in participants aged over 50 years but not in younger people between the ages of 35–49 years. In contrast to these studies, a study by Heng et al. [87] reported higher plasma concentrations of tocotrienols in younger people 30–34 years old, compared to people 50–54 years old after receiving TRF supplementations for six months. In study by Qureshi et al. [77], TRF doses at 125 mg/d, 250 mg/d, and 500 mg/d were given orally to healthy fed subjects (*n* = 11/dose), and dose-dependent increases in AUC and *C*_max_ were found. In a later study by Qureshi et al. [78], the safety and bioavailability of higher oral doses of 750 mg/d and 1000 mg/d of annatto-based tocotrienols in healthy fed subjects (*n* = 3/dose) were analyzed. This study showed a dose-dependent increase in plasma concentration (ng/mL) and plasma *t*_max_ of 3.33 and 4 h; elimination half-lives of 2.74 and 2.68 h for δ-tocotrienol. Similar results were reported for all other tocols, except for α-tocopherol. It was reported that a dose of δ-tocotrienol lower than 500 mg/d decreased the levels of serum total cholesterol, LDL-cholesterol, and triglycerides in a dose-dependent manner, but a higher dose of 750 mg/d increased the levels of these lipid parameters, compared to 250 mg/d [88]. Studies suggest that δ-tocotrienol has the unique dual biological property of inhibition (anti-inflammatory) and activation (pro-inflammatory), depending on its concentration [78,79,88,89,90]. The bioavailability of a mixture of α-tocotrienol, GT3, and DT3 was studied after administering a 300 mg capsule to fasted and fed healthy subjects (*n* = 8) and found the plasma *t*_max_ to be between 3 and 5 h for both fasted and fed subjects [57]. In another study, plasma *t*_max_ after administering two 300 mg/capsules of a mixture of γ-tocotrienol and δ-tocotrienol was found to be 5.64 ± 1.50 h and 4.73 ± 0.90 h, respectively [91]. When a dose of 450 mg of a rich fraction of barley oil versus a rich fraction of palm oil was administered to healthy subjects (*n* = 7), the area under the curve (0–24 h) of total (α-, β-, γ-, δ-) tocotrienols was significantly (2.6-fold) higher in the barley oil than in the palm oil [92].

A study by Abuasal et al. [93] compared the intestinal absorption kinetics and the bioavailability of GT3 and AT administered to rats. The oral bioavailability of AT (36%) was significantly higher than GT3 (9%), and AT showed higher intestinal permeability than GT3. These results indicate that the intestinal permeability could be a contributing factor for the higher bioavailability of α-tocopherol and suggests that enhancing the permeability of γ-tocotrienol would increase its oral bioavailability. There are findings suggesting that increasing the permeability of GT3 via new approaches, like solid lipid nanoparticles, leads to an increase in its bioavailability [94]. It was reported that co-administration of tocotrienols with lipids causes a delay in the rate of gastric emptying and this leads to an increase in tocotrienol’s solubility by stimulating the secretion of bile salts and phospholipids into the GI tract and therefore increasing the absorption and bioavailability of tocotrienols [95,96]. 

A self-emulsifying drug delivery system (SEDDS) has been used to achieve increased bioavailability of tocotrienols. GT3 dissolved in SEDDS versus commercial Tocovid was studied in vitro and in vivo in rats [97]. There was a 2-fold increase in the solubilization, higher cellular uptake in vitro, and a 2-fold increase in oral bioavailability for the SEDDS formulation [97]. Alqahtani et al. [98] also reported the bioavailability of γ and δ tocotrienols, administered to rats as SEDDS compared to commercially available UNIQUE E^®^ tocotrienol capsules, and the results showed that SEDDS increased the bioavailability. However, bioavailability showed a progressive decrease with increased treatment dose due to nonlinear absorption kinetics [98].

**Figure 3:**
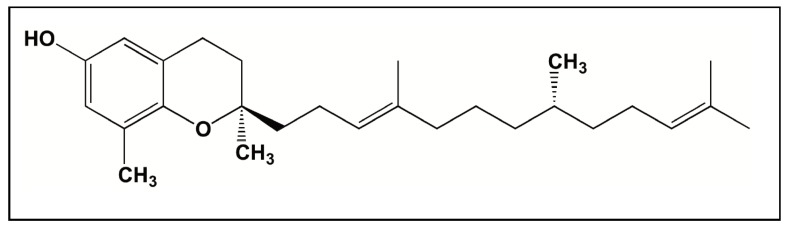
Chemical structure of δ-tocoflexol.

Collectively these studies show that tocotrienols respond differently at different doses in different populations. The major determinant for the limited bioavailability of the tocotrienols could be their limited binding to the transporter, ATTP, which is responsible for transporting the tocols out of the liver into the systemic circulation [99]. The key role of ATTP in regulating the pharmacokinetics of vitamin E has been elegantly demonstrated in several studies [61,99,100,101,102,103,104,105]. Previous studies have shown that there is a good linear relationship between relative affinity of tocols to ATTP and their biological activity [106]. These studies also demonstrate the need to conduct more randomized controlled trials with large sample sizes to better understand the bioavailability mechanisms and the therapeutic window of tocotrienols. Thus, it is highly desirable to develop a new vitamin E analog with an increased half-life and improved pharmacokinetic properties. This can be achieved by increasing the affinity of the analogs to ATTP, which may be responsible for maintaining the plasma levels of these compounds and also by increasing their permeability. Multiple studies [25,63,66] have recently reported the development of such analogs, the tocoflexols (Figure 3). The tocoflexols were designed, using computer aided techniques, to behave like tocopherols in terms of their bioavailability and like tocotrienols in terms of their biological activity. To that effect, tocoflexol has been shown to have an antioxidant activity and rate of cell uptake on a par with DT3 and GT3, but it has a greater ability to bind to ATTP than the tocotrienols and thus has the potential for improved bioavailability.

## 5. Conclusions and Future Directions

Given the increasing use of radioactive materials in healthcare as well as the number of nuclear reactors and the amplified nuclear terrorism risk, development of effective radioprotectors and radiomitigators that can be deployed immediately is the key to have a successful contingency strategy in case of unwanted/unexpected radiation exposures. Multiple studies have reported the mechanisms by which tocols exert their radioprotection. It has been suggested that tocols’ mechanism of protection from radiation-induced hematopoietic death involves cytokines and chemokines [8]. The importance of G-CSF induction on the mechanism of radioprotection of tocotrienols was demonstrated when the protective effects of GT3 were abrogated in irradiated mice treated with G-CSF antibodies [9,40,48]. It has also been shown that tocols reduce post-irradiation GI syndrome by decreasing IL-1β and IL-6 [107]. Some studies have shown that tocotrienols have a greater radioprotective effect than tocopherols because of their ability to inhibit HMG-CoA reductase [55]. The radioprotective effects of GT3 depend not only on its antioxidant properties but also on its ability to concentrate in endothelial cells [51]. A study by Loose et al. [47] investigated the gene expression profile in human epithelial cells after treatment with GT3, γ-tocopherol (GT), and AT for 24 h. GT3 was found to be more effective in modulating changes in gene expression than GT and AT, when a genome wide analysis was performed. Several genes were affected, including those responsible for cell cycle, cell proliferation, cell death, hematopoiesis, angiogenesis, and DNA damage. The poor bioavailability of the tocotrienols is a major limiting factor for their clinical use as radioprotectants and radiomitigators [66]. In this regard, the development of the tocoflexols represents a promising approach [25]. 

Low doses of ionizing radiation in medical treatments and occupational exposure results in high risk for cardiovascular diseases [108]. Long distance space missions are associated with exposure to galactic cosmic rays and solar particle events that can increase the risk for cataract, cancers, and cardiovascular diseases [109,110,111]. Because of their effectiveness and low toxicity, the tocols may prove to be effective to protect against low-dose radiation, but further research is required to establish their potential.

## Figures and Tables

**Table 1:** Acute Radiation Syndrome.

Acute Radiation Syndrome			
	Hematopoietic Sub-Syndrome	Gastro-Intestinal Sub-Syndrome	Neuro/Cerebrovascular Sub-Syndrome
Quantity of radiation	>2–3 Gy	5–12 Gy	10–20 Gy
Prodromal stage symptoms	Anorexia, nausea and vomiting	Anorexia, severe nausea, vomiting, cramps and diarrhea	Extreme nervousness and confusion; severe nausea, vomiting, and watery diarrhea; loss of consciousness; and burning sensations of the skin.
Latent Stage symptoms	Stem cells in bone marrow are dying, although patient may appear and feel well.	Stem cells in bone marrow and cells lining GI tract are dying, although patient may appear and feel well.	Patient may return to partial functionality.
Manifest Phase/Illness Phase symptoms	Anorexia, fever, and malaise. Drop in all blood cell counts occurs for several weeks.	Malaise, anorexia, severe diarrhea, fever, dehydration, and electrolyte imbalance.	Watery diarrhea, convulsions, and coma.
Recovery or death	Bone marrow cells will begin to repopulate the marrow.	>10 Gy radiation leads to death due to gastro-intestinal syndrome	No recovery is expected.
